# Valorization of Coal Fly Ash via a Novel SiFA/Chitosan/MnFe_2_O_4_ Composite Photocatalyst for Selective Oxidation of 5-Hydroxymethylfurfural to 2,5-Diformylfuran

**DOI:** 10.3390/molecules31213798

**Published:** 2026-10-22

**Authors:** Pedro Ângelo Miranda, Marta Monteiro, Claudia Santos, Alexandra Guedes, Bruno Valentim, Andreia F. Peixoto, Clara Rodrigues Pereira, Cristina Freire, Waldemar Maniukiewicz, Iwona Kuźniarska-Biernacka

**Affiliations:** 1 REQUIMTE/LAQV, Department of Chemistry and Biochemistry, Faculty of Science, University of Porto, Rua do Campo Alegre s/n, 4169-007 Porto, Portugal; suzanabunga@gmail.com (P.Â.M.); marta_monteiro11@hotmail.com (M.M.); clara.pereira@fc.up.pt (C.R.P.); acfreire@fc.up.pt (C.F.); 2 Earth Science Institute—Porto Pole, Department of Geosciences, Environment and Spatial Plannings, Faculty of Sciences, University of Porto, Rua do Campo Alegre s/n, 4169-007 Porto, Portugal; claudia.santos@gov.mb.ca (C.S.); aguedes@fc.up.pt (A.G.); bvvalent@fc.up.pt (B.V.); 3 Institute of General and Ecological Chemistry, Łódź University of Technology, Żeromskiego 116, 90-924 Łódź, Poland; waldemar.maniukiewicz@p.lodz.pl; 4 Department of Physical Chemistry, Faculty of Chemistry, University of Łódź, Pomorska 163/165, 90-236 Łódź, Poland

**Keywords:** photocatalysis, biomass valorization, semiconductive composites, coal ash, 5-hydroxymethylfurfural

## Abstract

The valorization of biomass-derived 5-hydroxymethylfurfural (HMF) and coal fly ash (CFA) offers opportunities for more sustainable chemical production. In this study, a silica-rich fraction recovered from CFA (SiFA) was combined with chitosan (CS) and manganese ferrite (MnFe_2_O_4_, MFO) to prepare a ternary SiFA/CS/MFO composite using co-precipitation and ionic gelation. The materials obtained at each stage of preparation were comprehensively characterized by X-ray fluorescence (XRF), Fourier-transform infrared spectroscopy (FTIR), Raman spectroscopy, X-ray diffraction (XRD), and scanning electron microscopy coupled with energy-dispersive X-ray spectroscopy (SEM–EDS). Under photocatalytic conditions in acetonitrile, the composite achieved 92.6% HMF conversion with 90.5% selectivity toward 2,5-diformylfuran (DFF). In contrast, thermal catalytic oxidation at 100 °C using tert-butyl hydroperoxide (tBuOOH) resulted in 99.7% HMF conversion but only 5% DFF selectivity. These results highlight the potential of fly-ash derived silica-based composites for selective photocatalytic valorization of a biomass-derived platform.

## 1. Introduction

The increasing environmental concerns associated with fossil fuel consumption, including climate change and pollution, have intensified the search for renewable and sustainable alternatives for the production of fuels and chemicals. Lignocellulosic biomass represents an abundant and renewable carbon resource capable of replacing a significant portion of fossil-derived chemicals [1,2]. Because of their environmental impact and finite availability, fossil fuels cannot sustainably meet the continuously growing global demand for energy and chemical products. Consequently, the valorization of biomass into high-value platform molecules has become a central focus of green chemistry and sustainable development [3].

Despite its robust and recalcitrant structure, lignocellulosic biomass can be efficiently converted into valuable intermediates through appropriate processing strategies. In particular, cellulose can be depolymerized into glucose and fructose, which serve as key precursors for the production of platform chemicals. Among these, 5-hydroxymethylfurfural (HMF) has been identified as one of the most promising biomass-derived platform molecules and was included in the list of top value-added chemicals from biomass in 2004, later updated by Bozell and Petersen in 2010 [4].

Due to its multifunctional structure, containing both aldehyde and hydroxymethyl groups, HMF can undergo a wide range of selective transformations, including oxidation, reduction, and ring-opening reactions, earning it the designation of a “sleeping giant” in biomass valorization [5,6]. In particular, DFF has attracted considerable attention in green catalysis due to its applications in the synthesis of furan-based polyesters, pesticides, antifungal agents, fluorescent materials, and advanced polymeric materials [5].

HMF can be selectively converted into several value-added chemicals. Among these, 2,5-diformylfuran (DFF) and 2,5-furandicarboxylic acid (FDCA) are considered key renewable alternatives to petroleum-derived monomers. Numerous catalytic systems have been investigated, including photocatalysts, electrocatalysts, and noble-metal catalysts. While very high conversions or yields have been reported, many of these systems rely on expensive metals, harsh conditions, or complex syntheses. For example, photooxidation of HMF to DFF has been achieved using composites of CdS quantum dots and the metal organic framework MIL-101, reaching a DFF selectivity of 50% at 30% HMF conversion under visible light irradiation for 5 h [5]. Electrochemical oxidation of HMF to FDCA has been achieved using Pt/CuO@CF electrocatalysts composed of Pt nanoparticles dispersed on CuO nanowires, affording FDCA yield and selectivity above 95% over an HMF concentration range of 10–100 mM while maintaining excellent stability over 15 consecutive cycles [7]. Similarly, catalytic oxidation using Ru-MnO_2_ composites has delivered FDCA yields up to 91% after 12 h at 100 °C [8], whereas gold nanoparticles (size 4–40 nm) supported on activated carbon have achieved 90% FDCA yield in the presence of NaOH at 70 °C under 10 bar O_2_ within 4 h [9].

However, conventional HMF oxidation processes typically rely on noble-metal catalysts and/or harsh reaction conditions, such as strong oxidants, elevated temperatures, and high pressures. These approaches present significant drawbacks, including high production costs, limited catalyst sustainability, and environmental concerns [10,11]. Therefore, the development of efficient, environmentally benign, and economically viable catalytic strategies for the selective oxidation of HMF remains a critical challenge in biomass conversion research.

Among semiconductors, manganese ferrite (MnFe_2_O_4_, MFO) has attracted increasing attention because of its narrow band gap, magnetic recoverability, chemical stability, and ability to activate molecular oxygen under light irradiation. However, its photocatalytic performance is often limited by nanoparticle aggregation and rapid charge-carrier recombination. Immobilization on suitable supports and the incorporation of dispersing agents represent possible strategies for addressing these limitations by influencing particle distribution. In addition to catalytic functionality, the selection of support materials is important from a sustainability perspective, particularly when considering the cost and environmental implications of catalyst preparation. In this context, the valorization of industrial by-products as catalyst supports offers an opportunity to integrate waste utilization into the development of photocatalytic materials. In this study, we investigate the selective oxidation of HMF using a multifunctional composite comprising MFO semiconductor nanoparticles immobilized within a chitosan (CS) matrix and supported on a silica-rich fraction of coal fly ash (SiFA). The main novelty lies in the integration of a waste-derived inorganic support, a biopolymer-based binding and dispersing phase, and a magnetic semiconductor within a single photocatalytic material. In this composite, SiFA serves as the support, while CS provides a matrix for binding and distributing MFO particles. This approach combines the utilization of an industrial solid-waste-derived material with the development of a composite photocatalyst for the selective conversion of a biomass-derived platform molecule. To the best of our knowledge, the use of the silica-rich fraction recovered from coal fly ash as a support for selective photocatalytic conversion of HMF to DFF was not reported previously. The study examines the effects of catalyst dosage, solvent, and substrate concentration on the photocatalytic reaction and includes a preliminary assessment of catalyst reuse, together with a comparison between photocatalytic and thermal catalytic oxidation. The results contribute to understanding the catalytic behavior of this particular material combination and demonstrate an approach that integrates industrial by-product valorization with the photocatalytic conversion of a renewable platform molecule into a value-added chemical.

## 2. Results and Discussion

### 2.1. Characterization of Materials

Table 1 lists the XRF analysis results for the SiFA, SiFA/CS, and SiFA/CS/MFO samples. SiFA exhibits the highest Si and Al contents. Following the introduction of chitosan (SiFA/CS), the Si and Al contents decrease from 27.4 to 26.2 wt% and from 12.1 to 10.4 wt% respectively. This reduction is likely caused by partial leaching induced by acetic acid during the preparation process. Consequently, the relative Fe content increases from 5.9 wt% in SiFA to 7.2 wt% in the SiFA/CS material. This Si leaching has previously been observed for blast furnace slag, where complete dissolution of Si occurred within 30 min in 5% acetic acid at room temperature [12]. Similarly, a significant decrease in Al content, from 21.2% to 6.2%, was reported after treating fly ash with acetic acid (pH 3.2–4.0) [13].

Further modification of SiFA/CS throughout the incorporation of MFO leads to substantial increase in the Mn content, from trace amounts to 6.4 wt%, and in the Fe content, from 7.2 wt% to 18.3 wt% in the resulting SiFA/CS/MFO composite. After correcting the Fe content for the Fe originally present in SiFA/CS, the calculated Fe/Mn ratio is 1.7, reasonably close to the theoretical value of 2.0 for MFO. This result is consistent with the presence of Fe- and Mn-containing material in proportions approaching the nominal composition of MFO.

**Figure 1:**
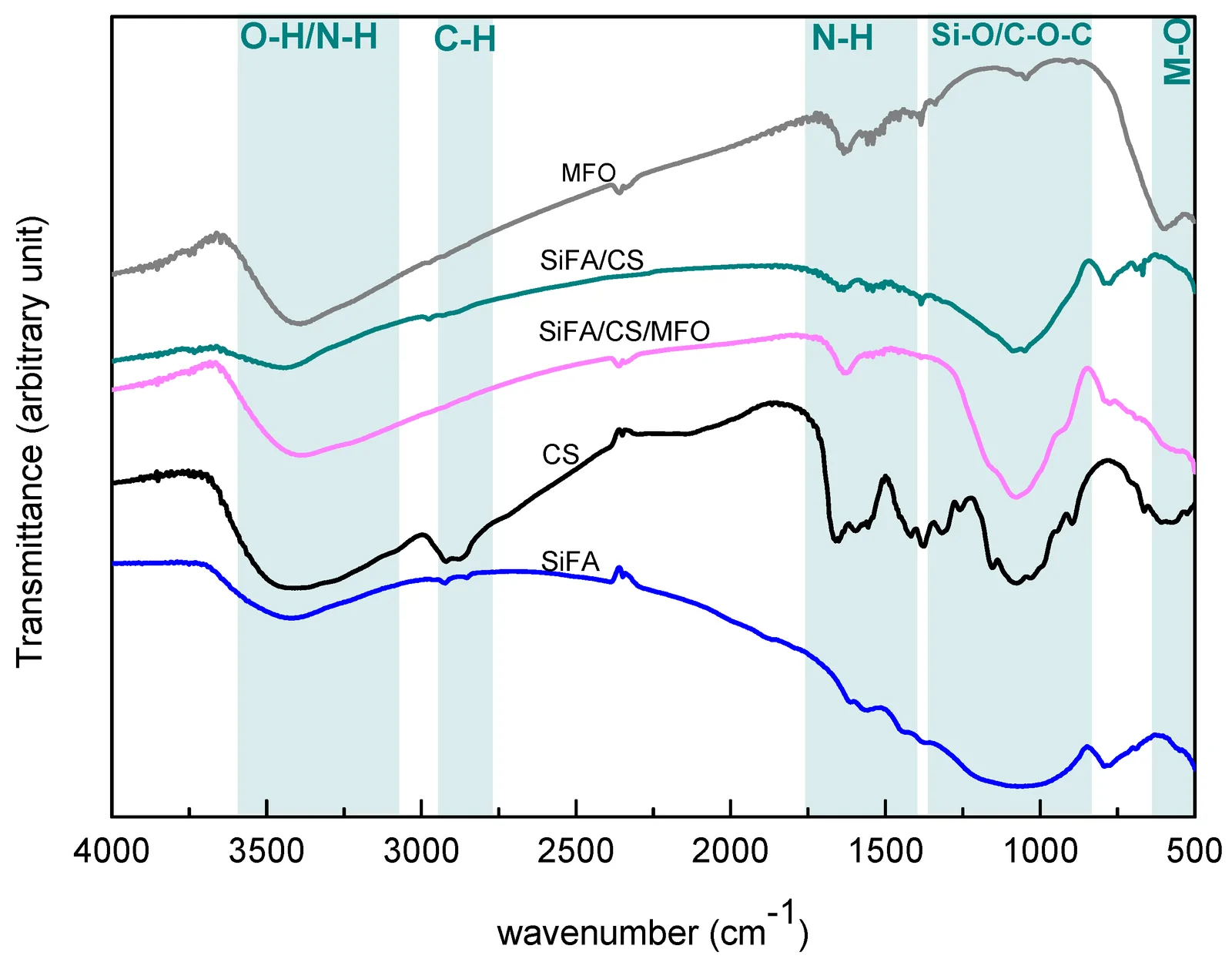
FTIR spectra of pristine CS, SiFA, MFO nanoparticles, and SiFA-based composite materials in 4000–500 cm^−1^ region.

The presence of functional groups and the nature of the chemical bonds in all prepared materials are investigated by FTIR spectroscopy. The FTIR spectra of pristine CS, MFO and SiFA, together with those of the SiFA/CS and SiFA/CS/MFO composites, are presented in Figure 1.

The FTIR spectrum of CS exhibits characteristic bands associated with carbohydrate, amide, and amine groups. A broad band in the 3750–3000 cm^−1^ region is attributed to the overlapping O–H stretching vibration (~3450 cm^−1^), arising from hydrogen-bonded hydroxyl groups of adsorbed water and carbohydrate ring (from CS), and N–H stretching vibration (~3240 cm^−1^) [14].

Weak bands at 2925 and 2875 cm^−1^ are assigned to C–H stretching vibrations. The amide I and II bands, corresponding to C=O stretching and N–H bending vibrations of the residual amides in CS, are observed at 1660 and 1595 cm^−1^, respectively [14]. In addition, C–N stretching vibrations appear in the 1190–920 cm^−1^ region and overlap with the carbohydrate ring vibration, which is observed as a band at 1078 cm^−1^ [15,16].

The interaction between CS and TPP in the SiFA/CS material is evidenced by a decrease in the intensities of the O–H and N–H stretching bands (~3450 cm^−1^ and ~3240 cm^−1^, respectively), together with the appearance of new bands at 1643 and 1545 cm^−1^. These spectral changes are consistent with the formation of an ionically cross-linked CS network [14]. The same characteristic bands are also observed for the SiFA/CS/MFO composite. However, the band at 1643 cm^−1^ in SiFA/CS shifts to 1639 cm^−1^ upon MFO incorporation, suggesting an interaction between the ferrite nanoparticles and the CS matrix.

The FTIR spectrum of pristine MFO exhibits a characteristic band at 605 cm^−1^, which is assigned to metal–oxygen stretching vibrations at tetrahedral sites [17]. In addition, a broad band in the 3550–3050 cm^−1^ region is associated with the O–H stretching vibrations of surface hydroxyl groups and physisorbed water molecules. Weak bands at 1629 and 1538 cm^−1^ are attributed to NH_2_ bending vibrations, indicating the presence of residual MIPA that is not completely removed during the washing step [18]. The characteristic metal–oxygen band at 605 cm^−1^ is not clearly distinguishable in the SiFA/CS/MFO spectrum because it overlaps with the low-wavenumber adsorption bands of the CS matrix. This overlap prevents unambiguous identification of MFO by FTIR. The weak or unresolved MFO band may be associated with the contribution of MFO to the composite spectrum, possible spectral overlap, or particle-size effects [19]; however, these factors cannot be distinguished conclusively from the available FTIR data.

**Figure 2:**
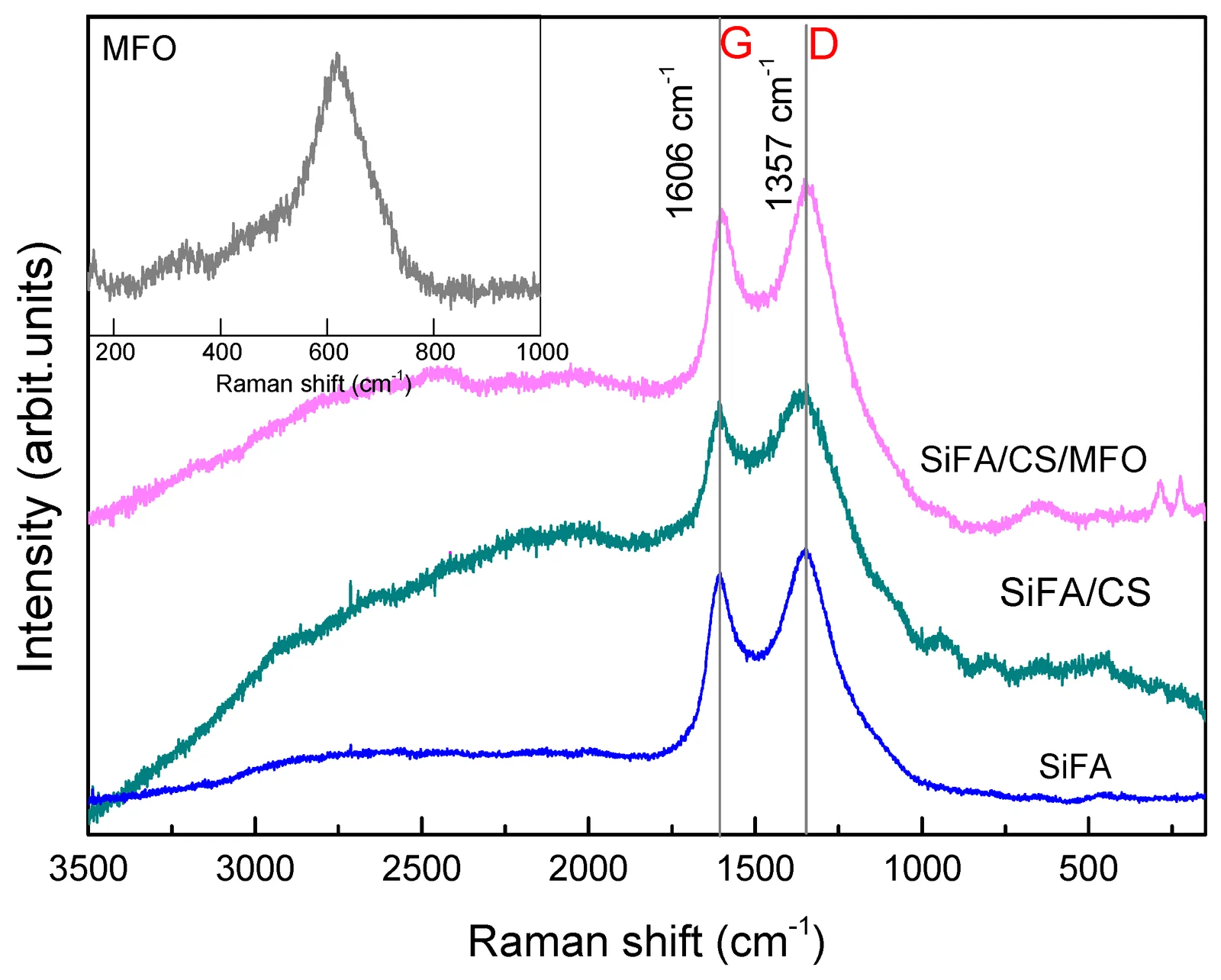
Raman spectra of the SiFA, SiFA/CS and SiFA/CS/MFO materials: MFO low-energy region; Insert: high-energy region.

The Raman spectra (3500–150 cm^−1^) of all prepared materials are shown in Figure 2. The Raman spectra of the SiFA-containing samples exhibit carbon-related bands, indicating the presence of residual carbonaceous material in the SiFA fraction [20,21,22]. The prominent bands observed near 1350 and 1590 cm^−1^ correspond to the D and G bands, respectively. Their spectral features, particularly the broad full width at half maximum (FWHM) and the pronounced asymmetry of the D band, suggest the presence of structurally diverse carbonaceous species associated with unburned carbon [21].

Quartz in the SiFA-containing samples is evidenced by the presence of a broad band around 470 cm^−1^, attributed to the symmetric Si–O stretching mode, along with weak bands in the 350–410 cm^−1^ region arising from lattice vibrational modes of quartz. Low-intensity bands near 650 cm^−1^ can be assigned to asymmetric SO_4_^2−^ bending vibrations, confirming the presence of other minerals in the samples, particularly in pristine SiFA [21].

In the SiFA/CS and SiFA/CS/MFO composites, characteristic Raman bands of CS and TPP occur at 1375 and 1461 cm^−1^, corresponding to CH_2_ deformation and C–H rocking vibrations. The 899 cm^−1^ band in the SiFA/CS composite is assigned to the symmetric stretching mode of the various P–OH groups. However, the CS-related bands are strongly overlapped by the D band of SiFA residual unburned carbon. The presence of a broad band in the region 860–1010 cm^−1^ is consistent with the formation of a CS–TPP cross-linked network [23].

The Raman spectrum of pristine MFO shows its most intense band at 625 cm^−1^, which is attributed to symmetric stretching of oxygen atoms at tetrahedral sites, along with less intense modes at 335 and 162 cm^−1^, characteristic of octahedral sites [18,19]. For the SiFA/CS/MFO composite, the band observed at 655 cm^−1^ may result from the overlap between the sulfate band originating from SiFA with the characteristic MFO band.

The presence of the characteristic MFO Raman band in SiFA/CS/MFO cannot be unambiguously confirmed. It has been reported that the intensity of the strongest MFO band increases with increasing particle size. Such an increase is not observed in the present study. When the particle size is small, the corresponding vibrational modes are weak, leading to low band intensity [19]. This observation is in agreement with the conclusions drawn from FTIR analysis.

**Figure 3:**
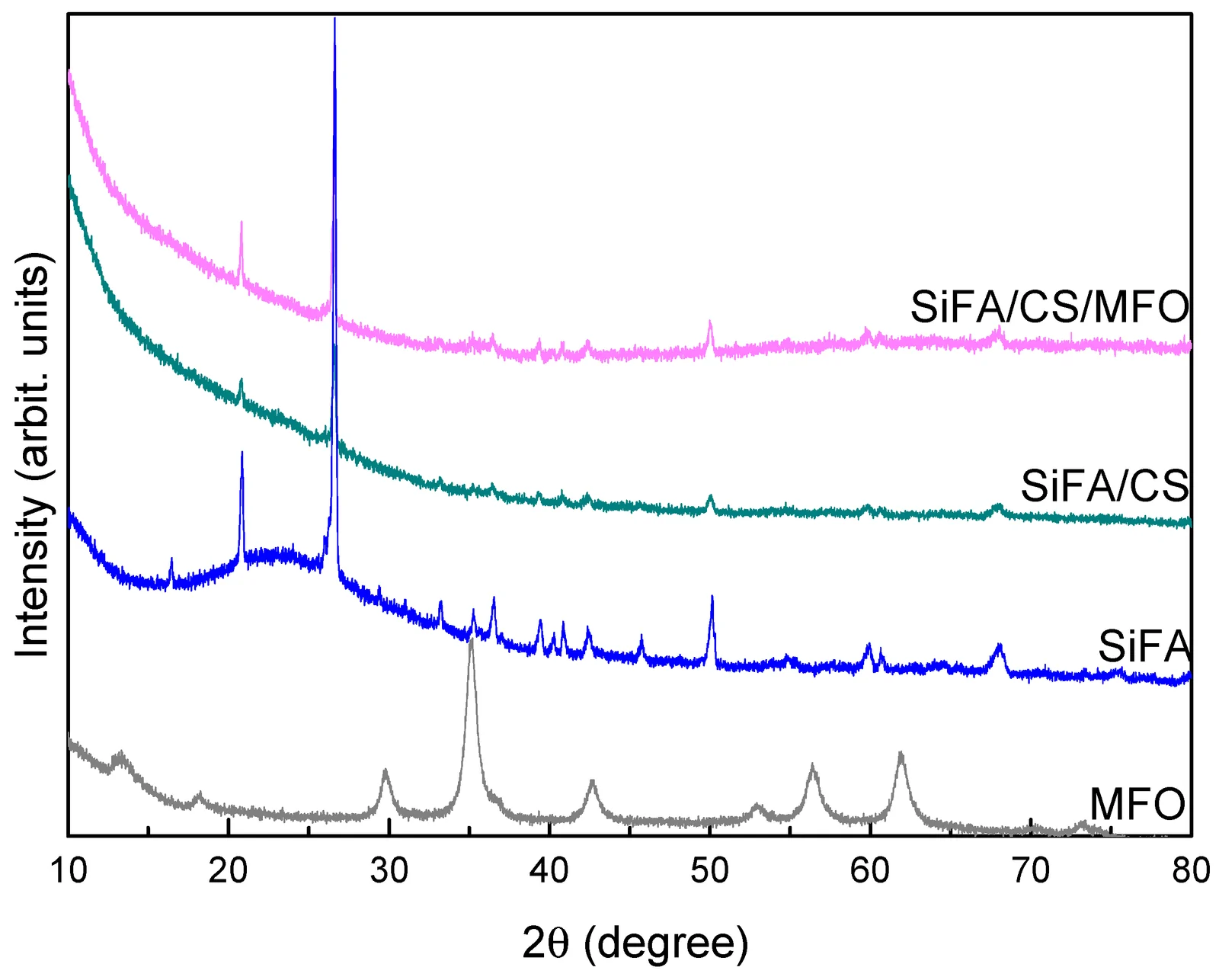
XRD patterns of pristine MFO, SiFA and composites SiFA/CS and SiFA/CS/MFO.

Figure 3 shows XRD patterns of all prepared materials. The main crystalline components of SiFA and the SiFA-based materials are quartz (SiO_2_), with diffraction peaks at 2θ = 20.85°, 26.639° and 50.138° for the (100), (101) and (112) reflections, respectively, and mullite (Al_6_Si_2_O_13_), with diffraction peaks at 2θ = 25.971°, 26.267° and 40.874° for the (120), (210) and (121) reflection lines, respectively. In addition, a broad diffraction peak is present between 15° and 35°, indicating that amorphous silica is also present in the materials [24]. The XRD pattern of pristine MFO, which was synthesized and characterized separately before being incorporated into the SiFA/CS matrix, exhibits diffraction peaks at 2θ—18.1°, 29.7°, 35.1°, 42.6°, 52.9°, 56.4°, and 62.0°—corresponding to diffraction planes (111), (220), (311), (400), (422), (511) and (440), respectively, characteristic of manganese ferrite with a spinel structure (JCPDS no. 10-0319) [18,25]. The crystallite size of pristine MFO was estimated to be 10.4 nm from the most intense reflection (311) using the Debye–Scherrer equation [18].

The SiFA/CS/MFO composite was subsequently prepared by incorporating this previously characterized MFO into the SiFA/CS matrix. However, the characteristic MFO reflections are not clearly resolved in the XRD pattern of the final composite. Their absence may be associated with the relatively low contribution of MFO to the overall diffraction pattern, overlap with reflections from other components, and/or changes in the diffraction features associated with the incorporation process. Similar peak broadening associated with decreasing crystallite size has been reported for MFO prepared under different synthesis conditions [19,26]. Nevertheless, the absence of clearly resolved MFO reflections does not allow the crystallite size or spatial distribution of MFO in the final composite to be established from XRD alone.

Complementary XRF analysis (Table 1) shows an Fe/Mn ratio reasonably close to the theoretical ratio of 2:1 for MFO supporting the presence of iron and manganese in proportions consistent with the nominal MFO composition. However, the available characterization does not independently confirm the persistence of the MFO crystalline phase or its dispersion in the final material. The characteristic crystalline phases of SiFA remain unchanged following composite formation.

**Figure 4:**
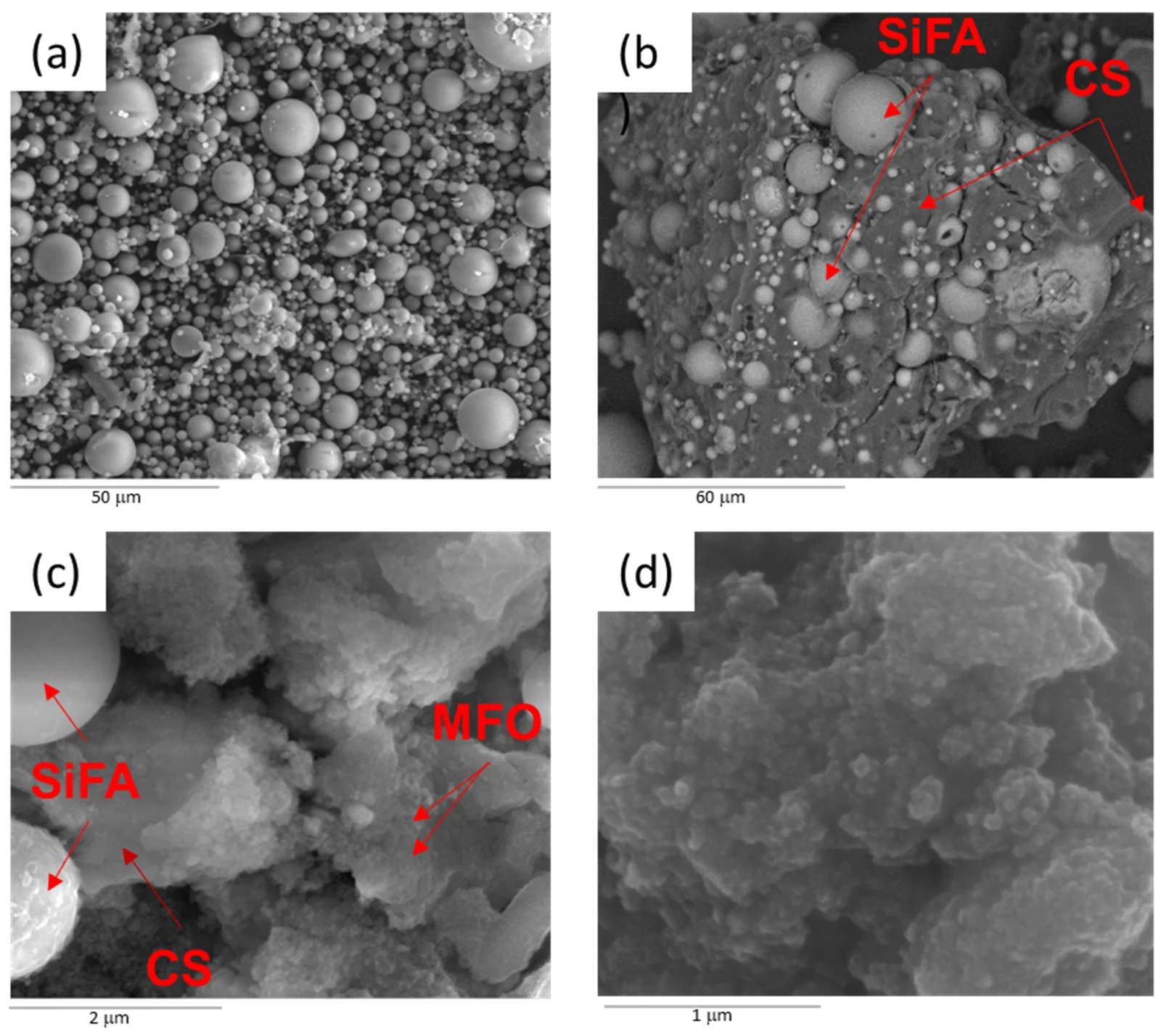
SEM images of (**a**) SiFA, (**b**) SiFA/CS, (**c**) SiFA/CS/MFO and (**d**) MFO particles.

The surface morphology of the samples was examined by SEM, while their elemental composition was determined by EDX. Representative SEM micrographs of the SiFA, SiFA/CS, MFO, and SiFA/CS/MFO materials are presented in Figure 4.

The SiFA fraction consists of typical aluminosilicate glassy cenospheres (Figure 4a). After the modification with CS, the spherical SiFA particles become coated with a chitosan layer (Figure 4b). Nanostructured features are observed in the SiFA/CS/MFO composite (Figure 4c). Although these features may be consistent with the presence of incorporated particles, SEM imaging alone does not establish their identity as MnFe_2_O_4_. The bare MFO particles exhibit nanoscale dimensions (Figure 4d), which is consistent with dimensions indicated by the Raman and XRD analyses.

The EDX analysis indicates that the major constituents of the SiFA cenospheres are Si (25.4 wt%) and Al (12.3 wt%), together with smaller amounts of C (4.8 wt%) and Fe (3.9 wt%), in good agreement with the XRF results. Following CS modification, the carbon content increases to 46.1 wt%, reflecting the formation of the chitosan coating on the cenosphere surface. The increase is accompanied by a corresponding decrease in the relative Si and Al contents to 3.1 wt% and 2.2 wt%, respectively.

The incorporation of MFO into the SiFA/CS system results in the appearance of Mn, which is below the detection limit (<1 wt%) in the pristine SiFA but reaches 7.4% in the SiFA/CS/MFO composite. These compositional changes are in good agreement with the XRF data and support the successful deposition of MFO onto SiFA/CS, thereby supporting the successful fabrication of the composite materials.

Overall, the combined characterization results provide evidence for the presence of Fe- and Mn-containing material in the SiFA/CS/MFO composite. The Fe/Mn ratio obtained by XRF is reasonably close to the theoretical ratio for MFO, while EDX confirms the presence of Mn in the final material. However, the characteristic MFO reflections are not clearly resolved in the composite XRD pattern, and the FTIR and Raman spectra do not allow unambiguous identification of the MFO phase. Therefore, although the results are consistent with the incorporation of MFO-derived material into the SiFA/CS matrix, the presence of crystalline MFO and its spatial distribution within the final composite have not been conclusively established. Further characterization using TEM/HRTEM and STEM-EDS mapping would be required to provide more direct evidence of the phase identity and distribution of the Fe- and Mn-containing material.

### 2.2. Catalytic Performance and Adsorption Studies

Since adsorption can contribute to the apparent removal of HMF, adsorption studies were performed prior to the catalytic experiments, to distinguish adsorption from (photo)catalytic degradation. The UV-Vis spectrum of an aqueous HMF solution exhibits two characteristic absorption bands at 229 and 284 nm. The absorption band at 284 nm is intense and well resolved, making it suitable for quantitative spectrophotometric analysis. Therefore, HMF concentration was determined by monitoring absorbance at 284 nm.

The stability of the standard HMF solutions was evaluated over a period of 10 days, during which no significant spectral changes are observed. The prepared materials (SiFA, SiFA/CS, MFO, and SiFA/CS/MFO) exhibit negligible HMF adsorption (<10%) after 300 min of contact time. Therefore, adsorption kinetics were not investigated further.

The photocatalytic activity of all prepared materials toward HMF oxidation is subsequently evaluated. The concentration of HMF and reaction products are monitored by GC for reactions carried out in ACN and by HPLC for reactions performed in water. Control experiments conducted under UV-A irradiation in the absence of a catalyst showed no measurable HMF oxidation after 300 min.

#### 2.2.1. Catalyst Dosage Effect

The effect of catalyst dosage on HMF conversion is investigated using the SiFA/CS/MFO catalyst, while all other reaction conditions are kept constant, including the HMF concentration (0.25 mg/mL), the water volume (100 mL), and the UV-A light source (15 W). In the absence of the catalyst, no HMF conversion is observed. Increasing the catalyst dosage from 0.01 to 0.5 mg/mL enhances HMF conversion from <5% to 38.1%. This improvement in HMF conversion is attributed to the greater number of available active sites and enhanced photon absorption provided by the higher catalyst loading.

At higher catalyst concentrations (1.0 mg/mL), increased light scattering and light shielding reduce the effective irradiation of the catalyst particles, resulting in a decrease in photocatalytic efficiency. This behavior was commonly observed for heterogeneous photocatalytic systems employing suspended powder catalysts [24]. Therefore, the optimal catalyst dosage is established as 0.5 mg/mL.

#### 2.2.2. Effect of Solvent and Catalyst Type

To examine the effects of the solvent and catalyst type, the conditions established in the catalyst dosage study are maintained, including the optimal catalyst loading (0.5 mg/mL) and HMF concentration (0.25 mg/mL). Acetonitrile (ACN) and water, differing mainly in polarity, viscosity and protic character [27,28], are selected as reaction media for the photocatalytic experiments.

**Figure 5:**
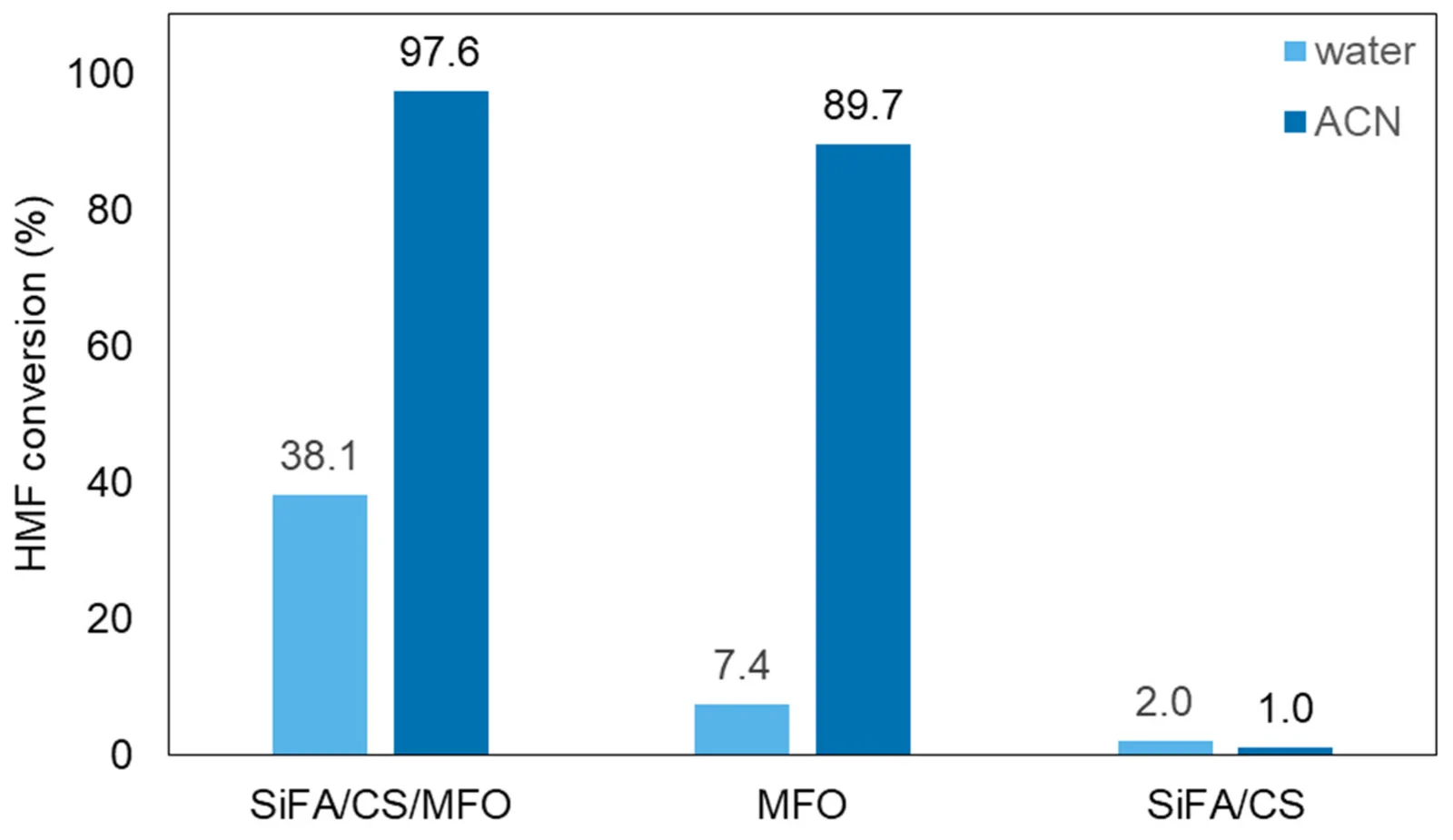
Effect of solvent on the conversion of HMF catalyzed by SiFA/CS/MFO, SiFA/CS, and MFO after 5 h of light irradiation.

In ACN, an aprotic solvent with polarity index of 8.40 and a density of 0.78 g/mL, all materials studied exhibit higher HMF conversion than in water. The SiFA/CS/MFO photocatalyst achieves the highest conversion (97.6%), followed by MFO (89.7%), whereas the SiFA shows no photocatalytic activity under the same reaction conditions (Figure 5). This trend is consistent with the findings of Zhang et al. [29] for Nb_2_O_5_ photocatalysts, who attributed the lower HMF conversion observed in water to competition between the solvent and reactants for access to the catalytic sites.

In water with a protic solvent with a polarity index of 12.10 and a density of 1.0 g/mL, the SiFA/CS/MFO photocatalyst achieves a 38.1% HMF conversion, whereas MFO shows only low conversion (<10%; Figure 5).

Although both catalysts demonstrate some activity toward HMF conversion, HPLC analysis detects no desired oxidation products, indicating that oxidative degradation of HMF predominates under these conditions rather than selective product formation. A similar trend was observed for poly(heptazine imides) doped with Fe, Co, Ni, or Cu, for which the desired products were negligible after 8 h of irradiation: 2,5-diformylfuran (DFF) was below the detection limit, and only trace amounts of 2,5-furandicarboxylic acid (FDCA) were detected [30].

The higher activity of the SiFA/CS/MFO composite compared with individual components suggests a cooperative effect among the constituent materials. SiFA may primarily act as a support, while CS may improve the dispersion of MFO particles and may contribute to interfacial charge transfer, thereby contributing to the enhanced photocatalytic performance [31]. MFO is likely to provide the photocatalytically active sites involved in HMF oxidation. However, under the applied reaction conditions, the oxidation predominantly results in non-selective degradation rather than selective formation of DFF.

#### 2.2.3. Effect of the Substrate Concentration

To investigate the effect of HMF concentration on catalytic performance and product selectivity, the solvent, catalyst dosage, irradiation conditions, reaction temperature and reaction time were kept constant, as established in the preceding optimization experiments. Photocatalytic reactions were conducted in ACN at room temperature with a catalyst dosage of 0.5 mg/mL. HMF concentrations of 0.05, 0.25 and 1.0 mg/mL were investigated, corresponding to catalyst:HMF mass ratios of 10:1, 2:1 and 1:2 respectively. The reaction mixtures were irradiated with a 15 W UV-A lamp for 5 h with a total volume of 100 mL under ambient air atmosphere.

At an HMF concentration of 0.05 mg/mL (catalyst:HMF mass ratio of 10:1) no products are detected above the analytical GC detection limit, indicating extensive degradation of HMF without detectable accumulations of oxidation products.

Increasing the HMF concentration to 0.25 mg/mL (catalyst:HMF mass ratio of 2:1) the SiFA/CS/MFO photocatalyst achieves 97.6% HMF conversion. As at the lower HMF concentration, no products are detected above the analytical GC detection limit. This indicates that HMF conversion occurs without detectable formation of the targeted products under these conditions. However, the formation of undetected intermediates or oligomeric products cannot be excluded, and the fate of the converted HMF cannot be established from the available analytical data.

**Figure 6:**
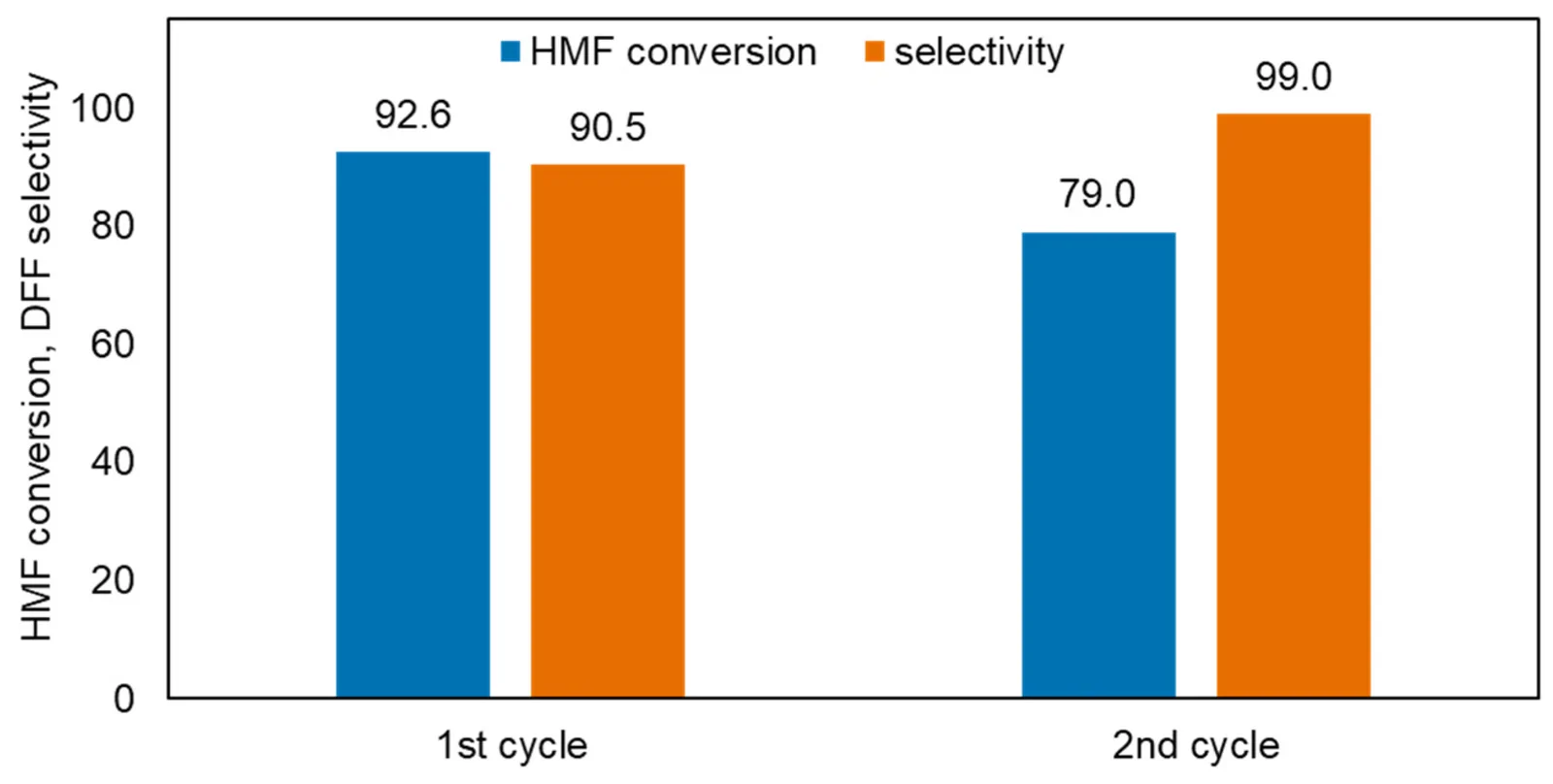
Stability of the SiFA/CS/MFO catalyst (1:2 catalyst to HMF ratio, in ACN).

At an HMF concentration of 1.0 mg/mL (catalyst:HMF mass ratio of 1:2; catalyst dosage = 0.5 mg/mL) HMF conversion decreases slightly to 92.6%, while DFF selectivity reaches 90.5% (Figure 6), and yield 83.8%. Under identical conditions, pristine MFO exhibited a lower HMF conversion (77.3%), with DFF as the only detected product.

Thus, the highest combined photocatalytic performance of the SiFA/CS/MFO composite was obtained using a catalyst dosage of 0.5 mg/mL and a HMF concentration of 1.0 mg/mL (catalyst:HMF mass ratio of 1:2), with ACN as the solvent, UV-A irradiation (15 W), a reaction time of 5 h, and at room temperature. Under these conditions, 92.6% HMF conversion and 90.5% DFF selectivity were achieved.

#### 2.2.4. Reusability of Catalyst

The reusability of the SiFA/CS/MFO photocatalyst is evaluated because it exhibits the highest photocatalytic performance in the initial reaction cycle. Under these conditions, the catalyst:HMF mass ratio is 1:2 in ACN. After the first reaction cycle, the catalyst is recovered by washing with water and ethanol and reused without any additional regeneration treatment. In the second reaction cycle, the HMF conversion decreases from 92.6% to 79.0%, while DFF remained the only detected product (Figure 6). These results demonstrate that the catalyst retained high selectivity towards DFF after one reuse cycle, although a decrease in HMF conversion was observed. The preservation of DFF selectivity suggests that the catalyst can be reused without a specific regeneration procedure under the investigated conditions [32].

The XRF results obtained for the SiFA/CS/MFO catalyst recovered after the second (reuse) cycle are summarized in Table 1. The results indicate a decrease in Mn content accompanied by a relative increase in Fe content: the Fe/Mn ratio increased from 1.7 for the fresh catalyst to 4.6 for the used catalyst. This change is consistent with a partial loss of Mn from the MFO phase during the photocatalytic reaction and/or the subsequent recovery and washing steps.

The normalized XRD patterns of the SiFA/CS/MFO catalyst before and after the second cycle (Figure S1, Supplementary Information) show that the main diffraction features of the composite remain largely unchanged after reuse. However, because the characteristic MFO reflections are not clearly resolved in the XRD pattern of as-prepared composite, the results do not allow persistence of the crystalline MFO phase after reuse to be independently confirmed.

Overall, the XRF and XRD results indicate a change in the elemental composition of the catalyst upon reuse, including a relative decrease in Mn content, while the main diffraction features of the composite remain largely unchanged. The decrease in Mn content may be associated with the reduction in catalytic activity observed in the second cycle; however, the available results do not establish a direct causal relationship or allow the precise deactivation mechanism to be determined conclusively. Furthermore, the reusability assessment comprised only one reuse cycle, and additional consecutive-cycle experiments would be required to evaluate the long-term stability of the catalyst more comprehensively.

The photocatalytic performance of the SiFA/CS/MFO composite is within the range reported for photocatalytic systems for the selective oxidation of HMF to DFF, including the Z-scheme heterostructure α-Fe_2_O_3_/Zn_0.5_Cd_0.5_S [33], Fe(III)-anchored porphyrin covalent organic frameworks (PCOF(Fe)-R, where R = –OMe, –OH, –CH_3_, –H, –Br) [34], ternary α/γ-MnO_2_/Cd_0.5_Zn_0.5_S heterojunction [35], TiO_2_-coated CsPbBr_3_ quantum dots [36], the composite of graphitic carbon nitride (g-C_3_N_4_) and V_2_O_5_ [37], the Zn_0.5_Cd_0.5_S/NaBiS_2_, S type heterojunction composite [38] and ZnIn_2_S_4_ [39]. The catalytic activity of SiFA/CS/MFO is comparable to that of cubic ZnS nanocrystals [40] and the g-C_3_N_4_/Bi_2_MoO_6_, heterostructured catalyst with 1:1 mass ratio g-C_3_N_4_:Bi_2_MoO_6_ [10]. However, a direct quantitative comparison of the catalytic performance is not possible because the reported studies were conducted under different experimental conditions, including light source and irradiation intensity, catalyst loading, initial HMF concentration, reaction volume solvent and reaction time. The experimental parameters of the literature systems are summarized in Table S1 (Supporting Information), allowing the reported performances to be evaluated in the context of their respective reaction conditions.

To provide a tentative rationale for the observed photocatalytic performance, the literature-reported electronic band structure of the photoactive MFO component was considered. In the SiFA/CS/MFO composite, MFO is assumed to serve as the primary photoactive component. According to previous studies, MFO exhibits a reported band gap value of approximately 1.32 eV [41], with the valence band potential (1.18–1.24 V vs. NHE) [41,42] and conduction band potential (−0.45 V vs. NHE) [42]. These values are derived from the literature and have not been experimentally determined for the SiFA/CS/MFO composite investigated in this study. Consequently, they provide a tentative basis for discussing possible photocatalytic pathways rather than a direct description of the electronic structure of the prepared composite.

Based on the reported band-edge positions, the valence band potential of MFO is insufficient to generate ^●^OH radicals via water oxidation. In contrast, the reported conduction band potential (−0.45 V vs. NHE) [42] is more negative than the O_2_/^●^O^2−^ redox potential (−0.33 V vs. NHE), suggesting that the reduction of molecular oxygen to superoxide radicals is thermodynamically feasible.

Accordingly, the formation of ^●^O^2−^ species represents a plausible oxygen activation pathway during photocatalytic oxidation of HMF to DFF. This interpretation is consistent with previous studies on the photocatalytic oxidation of HMF to DFF, which demonstrated the oxygen dependence of the reaction and suggested superoxide radicals as key intermediates in the oxidation mechanism [34,43,44,45]. Nevertheless, UV-Vis diffuse reflectance spectroscopy and experimental determination of the band gap and band-edge positions were not performed in the present study. Furthermore, radical scavenging experiments and electron paramagnetic resonance (EPR) measurements were not conducted. Therefore, the proposed mechanism is based on literature-reported properties of MFO and previous mechanistic studies and should be regarded as a tentative interpretation rather than an experimentally verified mechanism for the SiFA/CS/MFO composite. In particular, the involvement of superoxide radicals as the dominant reactive species remains unconfirmed.

#### 2.2.5. Catalytic Performance of SiFA/CS/MFO at High Temperature with an Oxidant

For comparison with photocatalytic oxidation, the fabricated materials are further evaluated as conventional heterogeneous catalysts for HMF oxidation in the presence of tert-butyl hydroperoxide (tBuOOH) in acetonitrile at 100 °C. Control experiments, performed using either tBuOOH alone or SiFA/CS/MFO alone result in less than 5% HMF conversion, with no detectable reaction products.

**Figure 7:**
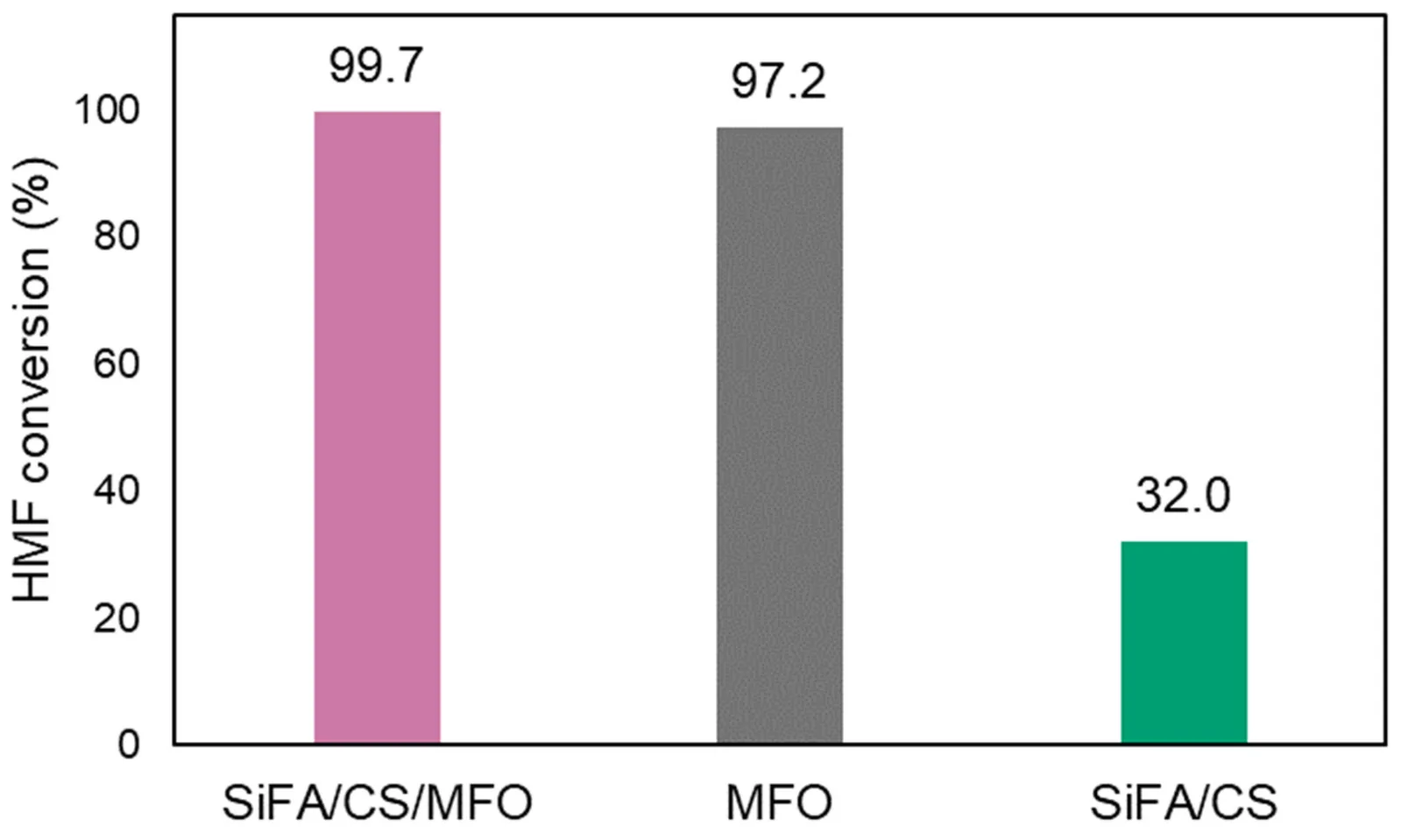
Conversion of HMF in catalytic oxidation at high temperature (100 °C).

Among the investigated materials, the SiFA/CS/MFO composite exhibits the highest catalytic activity, achieving 99.7% HMF conversion after 12 h. The MFO component alone also shows high catalytic activity, reaching a comparable HMF conversion of 97.2%, whereas SiFA exhibited significantly lower activity (32%); see Figure 7. Despite the high conversion values, both SiFA/CS/MFO and MFO show poor selectivity toward DFF (5.0%). Elevated reaction temperatures were reported to promote undesired side reactions during HMF oxidation, including the polymerization and further degradation of DFF, resulting in decreased selectivity toward the desired product [46,47].

To further evaluate the effect of temperature on selectivity, the reaction is performed at room temperature using the same catalytic system. Under these conditions, the DFF selectivity of SiFA/CS/MFO increases to 46.0%; however, the HMF conversion decreases significantly to 22.6%.

A direct comparison between photocatalytic and conventional catalytic oxidation using SiFA/CS/MFO is challenging because the reaction conditions differ significantly in terms of oxidant, temperature, and reaction time. Nevertheless, SiFA/CS/MFO exhibits the highest activity among the investigated materials, under both reaction regimes, achieving HMF conversions of 92.6% and 99.7% under photocatalytic and conventional catalytic conditions respectively.

In contrast, the DFF selectivity differs considerably between the two systems, reaching 90.5% under photocatalytic conditions but only 5% in the presence of tBuOOH. This difference suggests that distinct oxidation pathways may operate under photocatalytic and thermal catalytic conditions. Under conventional catalytic conditions, the tBuOOH-based oxidation system appears to promote non-selective oxidation pathways, leading to the formation of by-products and subsequent degradation reactions rather than the selective DFF formation. Similar behavior was reported for TiO_2_-based catalytic systems [48].

The catalytic performance of SiFA/CS/MFO is further compared with previously reported Mn-containing catalysts [11,49,50,51], spent dry cell battery cathode material [46], and Ru-supported catalysts [52]. The comparative data are listed in the Supporting Information (Table S2). Reported catalysts exhibit HMF conversions ranging from 37 to 97%, which are lower than that achieved with SiFA/CS/MFO (99.7%), although they generally provide substantially higher DFF selectivity (>85% compared with 5% for SiFA/CS/MFO), under broadly comparable reaction conditions (80–120 °C).

## 3. Materials and Methods

### 3.1. Materials and Reagents

All reagents were used as received; manganese (II) chloride tetrahydrate (MnCl_2_ × 4H_2_O, ≥98%), iron (III) chloride hexahydrate (FeCl_3_ × 6H_2_O, ≥99%), hydrochloric acid (HCl, 37%) and acetic acid (C_2_H_4_O_2_, ≥99.8%) were purchased from Honeywell, Seelze, Germany. 1-amino-2-propanol, MIPA (C_3_H_9_NO, ≥93%), sodium triphosphate, TPP (Na_5_P_3_O_10_, ≥85%), 5-(hydroxymethyl)furfural, HMF (C_6_H_6_O_3_, >>99%), chitosan, CS ((C_6_H_11_NO_4_)_n_, >>93%), formic acid, FAc (HCOOH, ≥98%), ethanol (C_2_H_6_O, ≥99.8%), acetone (C_3_H_6_O, ≥99.5%), potassium bromide (KBr, ≥99.9%), tetradecane (C_14_H_30_, ≥99%), tert-butyl hydroperoxide solution 70 wt% in H_2_O (tBuOOH), acetonitrile, ACN (CH_3_CN ≥ 99.9%), and nujol (mineral oil) were purchased from Merck, Darmstadt, Germany. The CFA was sampled from a Portuguese Power Plant burning pulverized coal.

### 3.2. Preparation of the Materials

#### 3.2.1. SiFA Preparation

The SiFA was prepared as described elsewhere [53]. Briefly, the Fe-rich fraction was removed using low- and high-field magnets, while the carbon-rich fraction was removed by sieving, impaction, and elutriation.

#### 3.2.2. Synthesis of MnFe_2_O_4_ Nanoparticles (MFO)

The MnFe_2_O_4_ nanoparticles were prepared by the co-precipitation method as described elsewhere [18]. Briefly, MnCl_2_ × 4H_2_O was dissolved in 1 M HCl, while FeCl_3_ × 6H_2_O was dissolved in water. Both solutions were heated to 50 °C and then added to 3.0 M MIPA maintained at 100 °C. After cooling, the precipitate was magnetically separated, washed with deionized water until neutral pH was reached, and vacuum-dried for 3 h to obtain MFO.

#### 3.2.3. Preparation of SiFA/CS

SiFA/CS was prepared as described elsewhere [54]. Briefly, SiFA was dispersed in acetic acid and ultrasonicated. CS was then dissolved in the same solution, followed by the dropwise addition of TPP. The resulting solid (SiFA/CS) was separated, washed, and dried.

#### 3.2.4. Preparation of SiFA/CS/MFO

The SiFA/CS/MFO composite was prepared following the procedure used for SiFA/CS, using MFO synthesized and characterized separately beforehand. Briefly, SiFA, MFO, and CS were dispersed in 5% acetic acid and ultrasonicated. Subsequently, TPP was added dropwise. The resulting composite was magnetically separated, washed, and vacuum-dried.

### 3.3. Physicochemical Characterization

FTIR spectra were obtained on KBr pellets (Merck, spectroscopic grade) containing 0.2 wt% of the prepared material. A Jasco FTIR 460 Plus spectrometer, Tokyo, Japan was used. All spectra were collected at room temperature in the range of 400–4000 cm^−1^ using a resolution of 4 cm^−1^ and 64 scans.

X-ray powder diffraction (XRD) patterns were collected using a PANalytical X’Pert Pro MPD diffractometer in the Bragg–Brentano reflection geometry with CuKα radiation from a sealed tube (Malvern Panalytical Ltd., Royston, UK). Measurements were performed in the range of 2θ = 5–90° with a step size of 0.0167°.

Scanning electron microscopy coupled with energy dispersive X-ray spectroscopy (SEM-EDS) was performed using a high-resolution (Schottky) microscope (Quanta 400 FEG ESEM, Eindhoven, The Netherland) with X-ray microanalysis and electron backscattered diffraction analysis (EDAX Genesis X4M, EDAX Inc., Mahwah, NJ, USA) at Centro de Materiais da Universidade do Porto (CEMUP, Porto, Portugal). The samples were analyzed in powder form after coating with a thin gold film using the SPI module sputter coater equipment (Structure Probe, Inc., West Chester, PA, USA).

X-ray fluorescence (XRF) analysis was performed using an X-MET7500 portable analyzer, Oxford Instruments, Abingdon, UK.

For gas chromatography (GC) analysis, the centrifuged aliquots were directly injected into a Thermo Scientific Trace 1300 gas chromatograph equipped with a flame ionization detector (FID) and TG-5MS GC column (30 m length, 0.25 mm diameter inner, 0.25 µm film thickness).

The high-performance liquid chromatography (HPLC) analyses were performed on an UltiMate 3000 HPLC apparatus (Thermo Fisher Scientific, Rodano, Milan, Italy) with a 15 cm × 4.6 mm i.d. (5 µm) Ascentis C18 column (Merck, Darmstadt, Germany) equipped with a diode array detector (DAD 3000). The eluents were A: H_2_O/FAc (99.9:0.1) and B: ACN/H_2_O/FAc (95:5:0.1). After centrifugation of the collected aliquots, samples were diluted using a sample:H_2_O:FAc ratio of 20:79:1 before HPLC injection. DFF was identified by comparison of its retention time with that of authentic DFF standard.

### 3.4. Adsorption and (Photo)Catalytic Activity Evaluation

Adsorption assays were performed in the dark. In a typical experiment, 100 mL of HMF solution (c = 0.25 mg/mL) and 50 mg of material (c = 0.5 mg/mL) were introduced into the reactor (immersion well quartz reactor—PRL 3312, Photochemical Reactors Ltd., Reading, UK). Samples were collected at fixed time intervals, centrifuged, and analyzed by UV-Vis spectroscopy using an Agilent 8453 UV-Vis spectrometer (Agilent Technologies, Inc., Santa Clara, CA, USA) equipped with a diode array detector (quartz optical cell, 10 mm path length; spectral range of 200–800 nm).

The HMF removal percentage (%R) was calculated as follows:

%R = ((A_0_ − A_t_)/A_0_) × 100 (1)

where A0 and At are the absorbance values corresponding to λmax = 284 nm at the beginning of the adsorption process and at any time, respectively.

The photocatalytic oxidation of HMF was performed without a prior dark adsorption–desorption equilibration step. For the photocatalytic experiments, an appropriate amount of photocatalyst was added to a defined volume of solvent containing HMF (substrate). Different solvents and photocatalyst loading were investigated depending on the experimental conditions. The reaction mixture was irradiated with a OSRAM low-power UV-A lamp (315–400 nm) (OSRAM GmbH, Munich, Germany), electrical power 15 W and an emission maximum at 368 nm, for 5 h at room temperature. The lamp was positioned vertically at the center of the reaction vessel and immersed directly into the reaction mixture, as represented in Scheme S1 in Supplementary Information.

A blank experiment (photolysis) was performed under otherwise identical irradiation conditions in the absence of the photocatalyst. The progress of the reaction was monitored by GC when in ACN was used as the solvent, using tetradecane as an internal standard, and by HPLC for reactions performed in aqueous solutions.

The catalytic oxidation of HMF under dark conditions was performed in the presence of tert-butyl hydroperoxide (tBuOOH). In a typical experiment, 5 mg of catalyst was dispersed in 10 mL of ACN followed by the addition of 10 mg HMF and 0.1 mL of a 70 wt. % tBuOOH aqueous solution. The reaction mixture was stirred for 12 h at 100 °C, and the reaction progress was analyzed by GC.

For the re-use tests, after completion of the first photocatalytic cycle, the materials were recovered by filtration, washed with water and ethanol, vacuum-dried, and subsequently tested under the same experimental conditions.

The HMF conversion, and the DFF yield and selectivity were calculated according to the following Equations (2)–(4):

(2) $$C_{\mathrm{HMF}}=\frac{c_{\mathrm{HMF},0}-c_{\mathrm{HMF},t}}{c_{\mathrm{HMF},0}}\times 100\%$$

(3) $$Y_{\mathrm{DFF}}=\frac{c_{\mathrm{DFF}}}{c_{\mathrm{HMF},0}}\times 100\%$$

(4) $$S_{\mathrm{DFF}}=\frac{Y_{\mathrm{DFF}}}{C_{\mathrm{HMF}}}\times 100\%$$

where C_HMF_, Y_DFF_ and S_DFF_, are conversion of HMF, yield and selectivity of DFF, respectively. c_(HMF,0)_ is the initial concentration of HMF, and c_(HMF,t)_, and c_(DFF)_ are the concentrations after the reaction of HMF and DFF, respectively.

## 4. Conclusions

This work demonstrates the potential of a composite material (SiFA/CS/MFO) prepared from a silica-rich fraction recovered from coal fly ash, chitosan, and manganese ferrite for the photocatalytic oxidation of biomass-derived HMF to DFF. Under the investigated conditions, the SiFA/CS/MFO composite achieved 92.6% HMF conversion, 90.5% DFF selectivity, and an 83.8% DFF yield in acetonitrile after 5 h of UV-A irradiation.

The photocatalytic performance depended on the catalyst composition, catalyst-to-HMF mass ratio and solvent, with ACN providing substantially better performance than water. The composite showed better photocatalytic performance than its individual components, suggesting a beneficial combined effect of its constituents.

Thermal oxidation using tBuOOH resulted in 99% HMF conversion and 5% DFF selectivity and demonstrated different product distribution under respective reaction conditions. However, the differences in reaction temperature, reaction time, and oxidant use do not permit a direct comparison of intrinsic catalytic performance.

After reuse cycle, the SiFA/CS/MFO catalyst retained DFF as the only detected product, although HMF conversion decreased from 92.6% to 79.0%. Post-reaction XRF analysis revealed a relative decrease in Mn content, while XRD showed that the main diffraction features of the composite were largely preserved. These results suggest a possible relationship between compositional changes and reduced catalytic activity, although the deactivation mechanism and long-term stability remain to be established.

Overall, this study highlights the potential of integrating recycled fly ash derived silica-rich fraction with biopolymer and manganese ferrite components to develop photocatalysts for biomass valorization. Further work involving optical and electronic characterization, experimental identification of reactive species, and multi-cycle reusability tests is needed to clarify the photocatalytic mechanism and assess long-term catalyst stability.

## Figures and Tables

**Table 1:** XRF results (wt%) regarding elements of the initial and modified samples.

Sample	Selected Elements				Fe/Mn
	Si	Al	Fe	Mn	
SiFA	27.4	12.1	5.9	traces < 1	
SiFA/CS	26.2	10.4	7.2	traces < 1	
SiFA/CS/MFO	18.3	8.6	18.3	6.4	1.7 ^a^ (2.0)
SiFA/CS/MFO ^b^	18.7	7.5	21.2	3.0	4.6 ^a^

^a^ Ratio calculated from normalized Fe content in SiFA/CS material, in parentheses the theoretical value of Fe/Mn ratio for pristine MFO. ^b^ Used material. Analysis after the last catalytic cycle.

## Data Availability

Data are contained within the article and Supplementary Materials.

## Supplementary Materials

The following supporting information can be downloaded at https://www.mdpi.com/article/10.3390/molecules31213798/s1, Figure S1. Normalized XRD patterns of SiFA/CS/MFO sample before and after the last catalytic cycle; Figure S2. Emission spectrum of Lamp Osram UV-A; Scheme S1. Schematic representation of the photoreactor setup used; Table S1: Summary of Recently Reported Experimental Conditions and Performance in HMF-to-DFF Photocatalytic Oxidation; Table S2. Comparison of experimental conditions and results of catalytic oxidation of HMF to DFF.
