# Supplementary material for: Valorization of Coal Fly Ash via a Novel SiFA/Chitosan/MnFe2O4 Composite Photocatalyst for Selective Oxidation of 5-Hydroxymethylfurfural to 2,5-Diformylfuran
Source: Molecules. 2026 Oct 22;31(21):3798. doi: 10.3390/molecules31213798 (PMC13765126; doi:10.3390/molecules31213798)
Supplement: Supplementary file 1 [file molecules-31-03798-s001.zip › molecules-4618523-supplementary.pdf]

Valorization of Coal Fly Ash via a Novel SiFA/Chitosan/$MnFe_2O_4$ Composite Photocatalyst for Selective Oxidation of 5-Hydroxymethylfurfural to 2,5-Diformylfuran

Pedro Miranda[1,#], Marta Monteiro[1], Claudia Santos[2,$], Alexandra Guedes[2], Bruno Valentim[2], Andreia F. Peixoto[1*], Clara Rodrigues Pereira[1], Cristina Freire[1], Waldemar Maniukiewicz[3], Iwona Kuźniarska-Biernacka[1,4*]

1 REQUIMTE/LAQV, Department of Chemistry and Biochemistry, Faculty of Science, University of Porto, Rua do Campo Alegre s/n, 4169-007 Porto, Portugal; suzanabunga@gmail.com (PM); mar-ta_monteiro11@hotmail.com (MM); clara.pereira@fc.up.pt (C.R.P.); acfreire@fc.up.pt (C.F.)

2 Earth Science Institute – Porto pole, Department of Geosciences, Environment and Spatial Plannings, Faculty of Sciences, University of Porto, Rua do Campo Alegre s/n, 4169– 007 Porto, Portugal; clau-dia.santos@gov.mb.ca (C. S); aguedes@fc.up.pt (A.G.); bvvalent@fc.up.pt (B.V.)

3 Institute of General and Ecological Chemistry, Łódź University of Technology, Żeromskiego 116, 90-924 Łódź, Poland; waldemar.maniukiewicz@p.lodz.pl (W.M.)

4 University of Łódź, Faculty of Chemistry, Department of Physical Chemistry, Pomorska 163/165, 90-236 Łódź, Poland

# current address: Higher Institute of Educational Sciences, Uíge, Angola.

$ current address: Manitoba Geological Survey, 360-1395 Ellice Avenue, Winnipeg, MB R3G 3P2, Canada

* Correspondence: iwona.kuzniarska.biernacka@chemia.uni.lodz.pl (I.K.-B.); andreia.peixoto@fc.up.pt (A.F.P.)

Figure S1. Normalized XRD patterns of SiFA/CS/MFO sample before and after the last catalytic cycle

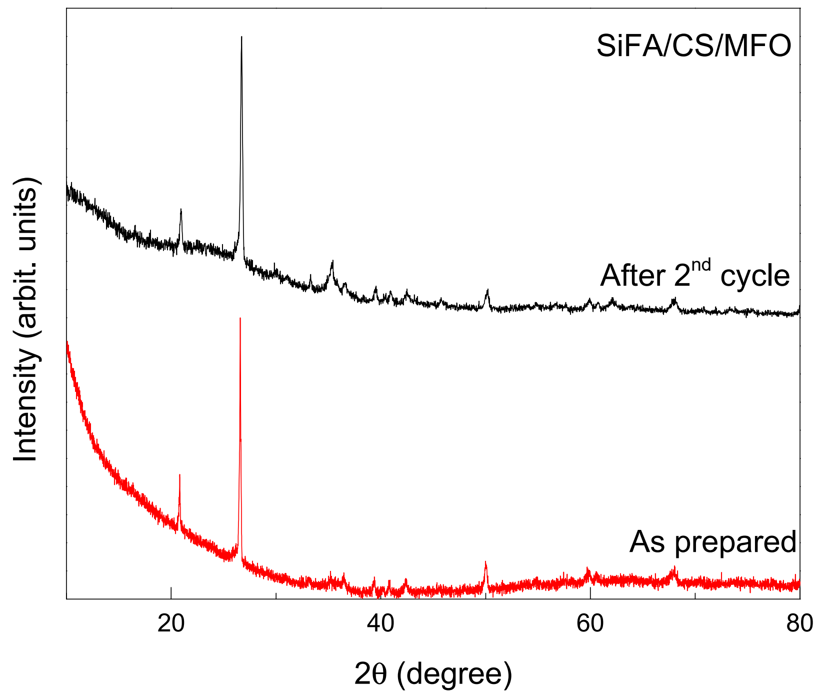

Figure S2. Emission spectrum of Lamp Osram UV-A

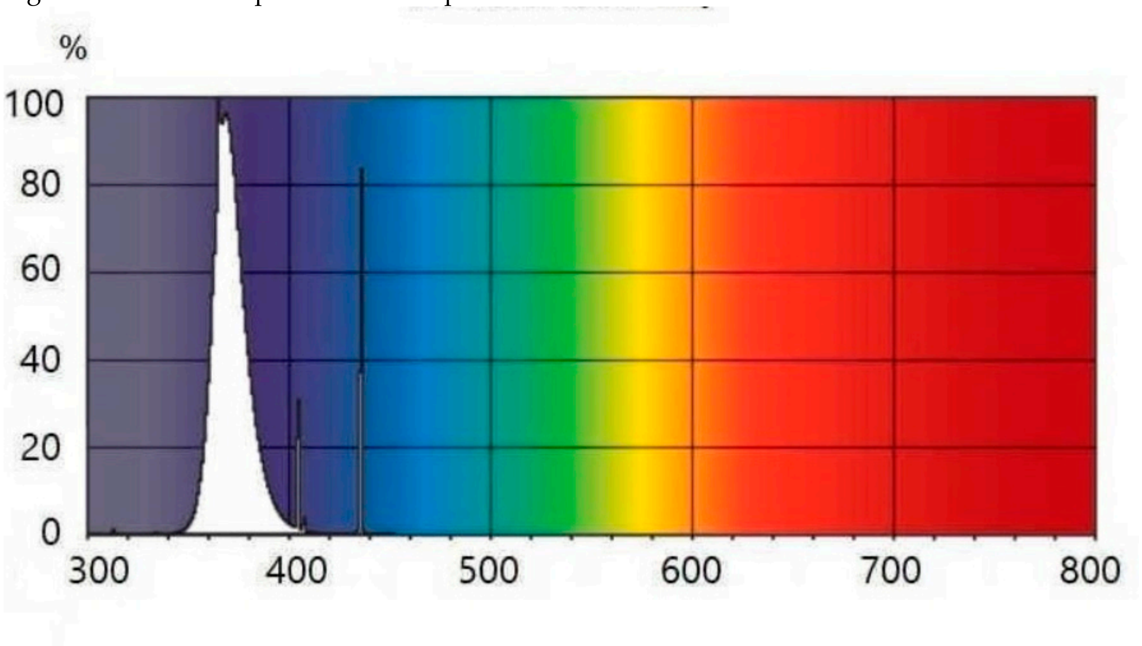

Scheme S1. Schematic representation of the photoreactor setup used

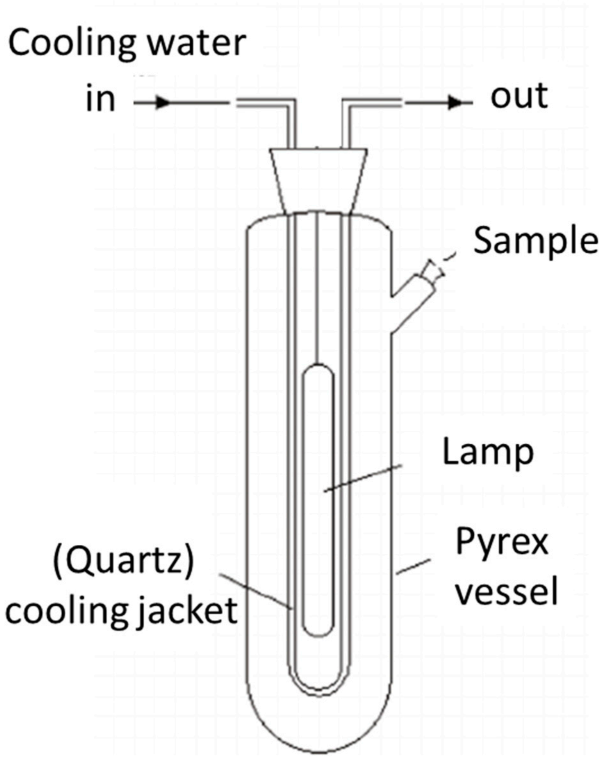

Table S1. Summary of Recently Reported Experimental Conditions and Performance in HMF-to-DFF Photocatalytic Oxidation.

catalyst	solvent	condition[a]	HMF:cat [mg/mg]	Time [h]	$\%C_{HMF}$[b]	$\%Y_{DFF}$[b]	$\%S_{DFF}$[b]	ref
c-ZnS[c]	ACN	7 W LED, λ_{max}=405.6 nm, RT, air,	1:25[d]	2	100		100	[1]
h-ZnS[c]	ACN			2			43	
α-$Fe_2O_3/Zn_{0.5}Cd_{0.5}S$[e]	water	12 W LED, λ_{max}=413 nm, Ar	1:0.16[d]	24	75		99	[2]
g-C_3N_4/Bi_2MoO_6[f]	ACN	10 W blue LED, λ_{max}=455 nm, air	1:0.03[d]	24	92.2		90.8	[3]
PCOF(Fe)-OMe[g]	water	300 W Xe lamp (λ> 400 nm, 3 mW cm^{-2}), O_2	1.4 [d]	6	-	90	-	[4]
PCOF(Fe)-OH[g]						84		
PCOF(Fe)-CH_3[g]						81		
PCOF(Fe)-H[g]						90.4		
PCOF(Fe)-Br[g]						79		
α/γ-$MnO_2/Cd_{0.5}Zn_{0.5}S$[h]	ACN	300 W xenon lamp,	1:1.6[d]	5	63		78	[5]
$CsPbBr_3$–TiO_2[i]	THF-Toluene	A solar simulador, 150 W short-arc Xe lamp, 50 °C	1:0.03[d, j]	22	80	72	91	[6]
V_2O_5/g-C_3N_4[k]	ACN+$PhCF_3$	300 W xenon lamp O2, flow 20 mL/min	1:4[d]	6	66.7	43.6	65.4	[7]
ZCS/NBS-7[l]	water	300 W xenon lamp, equipped with a 420 nm optical filter	1:0.18[d]	2	67.81		95.69	[8]
$ZnIn_2S_4$	water	50 W halogen lamp, air	1:16 [d]	5	43		68	[9]
SiFA/CS/MFO	ACN	UV-A 15 W, air	0:0.08	5	92.5		90.5	This work

[a] Reaction conditions: light source, temperature, RT-room temperature, atmosphere

[b] %C- converison of HMF, %Y- yield of DFF, %S- selectivity of DFF
[c] c-ZnS - cubic ZnS nanocrystals , *h*-ZnS -hexagonal ZnS nanocrystals
[d] calculated according to the data given in the literature
[e] Z-Scheme Heterostructure α-Fe_2O_3/$Zn_{0.5}Cd_{0.5}S$
[f] sonochemically synthesized g-C_3N_4/Bi_2MoO_6, heterostructured catalyst with 1:1 mass ratio g-C_3N_4:Bi_2MoO_6
[g] PCOF(Fe)-OMe, Fe(III)-anchored porphyrin covalent organic frameworks
[h] ternary α/γ-MnO_2/$Cd_{0.5}Zn_{0.5}S$ heterojunction prepared by hydrothermal synthesis
[i] in situ TiO_2 coated $CsPbBr_3$ quantum dots (QDs)
[j] mM ratio
[k] V_2O_5/g-C_3N_4 composite of graphitic carbon nitride (g-C_3N_4) and V_2O_5 fabricated by a simple calcination method.
[l] ZCS/NBS – the $Zn_{0.5}Cd_{0.5}S$/$NaBiS_2$ composite featuring S-type heterojunction prepared via a one-step alkaline hydrothermal method,

Table S2. Comparison of experimental conditions and results of catalytic oxidation of HMF to DFF

**catalyst**	**solvent**	**oxidant**	**HMF:cat [mg/mg]**	**Temp [°C]**	**Time [h]**	**%C_{HMF}[a]**	**%Y_{DFF}[a]**	**%S_{DFF}[a]**	**ref**
supported Ru [b]	MBE[c]	Air, 20 bar		80	1	54.5	54.5		[10]
	water	Air, 20 bar				55.1	28.9		
	ACN	Air, 20 bar				49.5	49.5		
$MnO_x/MnCO_3/Fe_2O_3$ (1:2)[d]	DMF	O_2, 0.8 Mpa	63:50[e]	100	2	45		97	[11]
$MnO_x/MnCO_3/Fe_2O_3$ (1:1) [d]	DMF	O_2, 0.8 Mpa				38		100	
$MnO_x/MnCO_3/Fe_2O_3$ (1:2) [d]	DMF	O_2, 0.8 Mpa				44		99	
V/Mn-1 [f]	ACN	O_2, atmospheric pressure	13:20[e]	80	12	47.6		97.2	[12]
MnO_x–A–U [g]	EtOH	O_2, 15bar	126:100[e]	120	4	78.3	75.6	96.6	[13]
BCM-2 [h]	Toluene	O_2 flow 10 mL/min	190:130[e]	110	15	37	35	99	[14]
	DMF			140	15	49	45	99	
	Water			100	15				
	ACN			82	15				
	DMSO			140	15	97	95	98	
3Mn/UZH0.5 [i]		None [j]			2	81.3	68.9	84.8	[15]
SiFA/CS/MFO	ACN	tBuOOH	1:4	100	12	99.7		5	This work
				ambient	12	22.6		46	

[a] %C- converison of HMF, %Y- yield of DFF, %S- selectivity of DFF
[b] covalent triazine frameworks-supported Ru
[c] methyl t-butyl ether
[d] different $MnO_x/MnCO_3/Fe_2O_3$ with diferente Mn:Fe molra ratio 1:2, 1:1 and 2:1
[e] calculated according to the data given in the literature
[f] V–Mn catalysts showed that the manganese to vanadium molar ratios of V/Mn − 1
[g] the manganese oxide synthesised using manganese acetate tetrahydrate and urea as precursor
[h] Spent Dry cell battery cathode material
[i] Mn-doped bimetallic UiO-66(Zr/Hf)-NH_2 piezoelectric photocatalyst, 3Mn/UZH0.5 catalyst (Zr/Hf = 1:1, 3.10 wt% Mn)
[j] Synergistic piezo-photocatalysis (light/ultrasound) process

References:

[1] Z. Wei, J. Zhang, M. Li, Y. Zhao, H. Wang, C. Li, Q. Qian, B. Han, Q. Li, J. Tai, Highly Selective Photocatalytic Conversion of 5-Hydroxymethylfurfural to 2,5-Diformylfuran by ZnS Nanocrystals, ChemSusChem, 18 (2025) e202501489.
[2] Y. Wang, C. Cai, Photocatalytic Oxidation of 5-Hydroxymethylfurfural to Selective Products by Noble Metal-Free Z-Scheme Heterostructures α-Fe2O3/Zn0.5Cd0.5S, ACS Catalysis, 15 (2025) 3451-3463.
[3] L.-Y. Jiao, Z.-L. Sun, W.-Y. Luo, Y.-Y. Xu, F. Wen, Y. Xu, Z. Li, W. Zhao, S. Liu, M. Ding, g-C3N4/Bi2MoO6 heterojunctions for enhanced visible light photocatalytic oxidation of biomass-derived 5-hydroxymethylfurfural to 2,5-diformylfuran under ambient conditions, Green Chemistry, (2026).
[4] Z. Cai, Y. Li, X.-X. Li, Y.-S. Feng, Substitution of functional groups on Fe(III)-anchored porphyrin covalent organic frameworks for the photocatalytic oxidation of hydroxymethylfurfural, Applied Catalysis A: General, 709 (2026) 120676.
[5] Y. Liang, F. Lin, C. Hu, Y. Cao, J. Cai, S. Yang, Q. Li, J. Wu, Z. Liu, Homojunction-mediated synergistic internal electric field interaction for the photocatalytic selective oxidation of 5-hydroxymethylfurfural to 2,5-dicarbonylfuran by α/γ-MnO2/Cd0.5Zn0.5S, Applied Surface Science, 713 (2025) 164084.
[6] V. Phuaran, M.S. Ahmad, K. Sumi, P.R. Pratama, Y. Akaishi, M. Takafuji, P. Vas-Umnuay, T. Kida, Interface-engineered CsPbBr3–TiO2 nanocomposites for efficient and selective photocatalytic conversion of HMF to DFF, Nanoscale, 17 (2025) 21132-21140.
[7] Y. Lin, D. Huang, C. Gong, Y. Zhou, Y. Wu, Simple synthesis of V2O5/g–C3N4 photocatalyst for the oxidation of biomass-derived 5-hydroxymethylfurfural, Research on Chemical Intermediates, 51 (2025) 1473-1489.
[8] S. Li, D. Wang, J. Chen, H. Zhang, F. Chen, Photocatalytic Selective Anaerobic Oxidation of 5-Hydroxymethylfurfural via Zn0.5Cd0.5S/NaBiS2 S-Scheme Heterojunction, ChemCatChem, 17 (2025) e00804.
[9] M. Umair, C.M. Pecoraro, F. Di Franco, M. Santamaria, L. Palmisano, V. Loddo, M. Bellardita, Enhanced aqueous photocatalytic selective oxidation of HMF using simulated sunlight: Comparison of the performance of different photocatalysts, Sustainable Materials and Technologies, 43 (2025) e01188.
[10] J. Artz, S. Mallmann, R. Palkovits, Selective Aerobic Oxidation of HMF to 2,5-Diformylfuran on Covalent Triazine Frameworks-Supported Ru Catalysts, ChemSusChem, 8 (2015) 672-679.
[11] L. Chen, F. Lou, H. Cheng, Z. Qi, Uniform heterostructured MnOx/MnCO3/Fe2O3 nanocomposites assembled in an ionic liquid for highly selective oxidation of 5-hydroxymethylfurfural, New Journal of Chemistry, 45 (2021) 12050-12063.
[12] J. Zhu, F. Cheng, F. Wang, S. Wen, X. Liu, Selective Oxidation of 5-Hydroxymethylfurfural to 2, 5-Diformylfuran Over a Vanadium Manganese Oxide Catalyst, Catalysis Letters, 152 (2022) 2280-2287.
[13] P. Pal, S. Saravanamurugan, Heterostructured manganese catalysts for the selective oxidation of 5-hydroxymethylfurfural to 2,5-diformylfuran, ChemCatChem, 12 (2020) 2324-2332.
[14] S.S. Ghadge, S.R. Bankar, V.H. Jadhav, Selective Oxidation of Biomass derived 5-Hydroxymethylfurfural (HMF) to 2,5-Diformylfuran (DFF) over Spent Dry cell battery cathode material (BCM-2), ACS Applied Engineering Materials, 2 (2024) 2651-2659.

[15] H. Xie, H. Ma, Z. Li, Y. Gu, F. Li, Y.-n. Wu, Green Piezo-Photocatalytic Oxidation of HMF to DFF Over Mn-Doped Zr/Hf-Based UiO-66 Frameworks Under Mild Conditions, ChemSusChem, 19 (2026) e202502033.
